# Editorial: Bacterial Post-translational Modifications

**DOI:** 10.3389/fmicb.2022.874602

**Published:** 2022-03-22

**Authors:** Valerie J. Carabetta, Julie Hardouin

**Affiliations:** ^1^Cooper Medical School of Rowan University, Camden, NJ, United States; ^2^Normandie Univ., UNIROUEN, INSA Rouen, CNRS, Polymers, Biopolymers, Surfaces Laboratory, Rouen, France; ^3^PISSARO Proteomic Facility, IRIB, Mont-Saint-Aignan, France

**Keywords:** acetylation, acylation, phosphorylation, lysine deacetylase and sirtuin, acetyltransferase, lipoprotein, post-translational modification (PTM), bacteria

The importance of post-translational modifications (PTMs) has been realized since the 1950s in eukaryotic cells. Since the turn of the century, we now realize that PTMs are ubiquitous in bacteria, and we must now turn our attention understanding the underlying function and purpose of these modifications. Modification of the side chains of critical amino acids in proteins may have important functional consequences, such as altering enzymatic activity, inhibiting DNA-binding activity, or altering protein conformation. Bacterial PTMs can be challenging to study, due to their relatively low stoichiometry, and inherently unstable nature. With the advancement of mass spectrometry-based proteomics workflows, detailed large-scale characterization of PTMs became possible. Today, there are thousands of identified PTMs, including phosphorylation, acetylation, and succinylation, distributed among hundreds of proteins in over 40 different bacterial species. However, the biological significance of the majority of such modifications still remains unknown. Moreover, the mechanisms of control of these processes and the enzymes involved are not completely understood.

This special topic issue focuses on newly identified PTMs, characterizing novel physiological functions of PTMs, and characterizing the enzymes involved. The first article in the collection characterizes the serine, threonine, tyrosine (STY) phosphosecretome in the opportunistic pathogen *Acinetobacter baumannii* (Massier et al.). The secreted phosphorylated proteins were characterized during two modes of growth, planktonic and biofilm. Interestingly, important virulent factors, such as those involved in adhesion, secretion and drug resistance were modified. The impact of a phosphorylated serine on the protein Hcp was also demonstrated by site-directed mutagenesis on the formation of a fully active type VI secretion system. Our next article explores the widely conserved, NAD^+^-dependent, lysine deacetylases, called sirtuins (Gallego-Jara et al.). The sirtuins have been well-characterized in eukaryotic systems, but their function and substrates in bacteria are not completely understood. This review provides an in-depth review of bacterial sirtuins in their physiology. An extensive phylogenetic study was performed that explores evolutionary relationships between sirtuins of different bacterial species and homologs. The next article looks at the process of acetylation/acylation from a mechanistic and structural biology perspective (Lammers). The current knowledge about the mechanisms of enzymatic regulation of lysine acetylation is discussed, including the known lysine acetyltransferases and classical Zn^2+^-dependent deacetylases. In addition, non-enzymatic acetylation, an important acetylation mechanism in bacteria, recent technological advances in the field and the physiological consequences of acetylation of well-characterized examples are discussed. The next article in the collection further explores the functional significance of the lysine acetylation of the histone-like protein HBsu in *Bacillus subtilis* (Luu et al.). Previously, it was found that HBsu contains seven acetylation sites, and that acetylation of key residues is required for normal chromosomal compaction. In this work, it was demonstrated that acetylation of HBsu also regulates the process of sporulation, and furthermore, influences the environmental resistance properties of mature spores. In this collection, Ma et al. review the PTMs that have been identified in oral bacteria, and what is known about their function. The oral bacteria live in the complex and constantly changing environment of the oral cavity. This review focuses on various modifications, including acetylation, phosphorylation, glycosylation, citrullination, succinylation, and glutathionylation that were identified in oral bacteria. The corresponding modifying enzymes and the potential physiological role of each PTM are discussed. The penultimate article focuses on the lipoprotein post-translational modifications (Smithers et al.). Lipoproteins are tethered to membranes by a lipid anchor and are abundant in bacteria. They often function as enzyme inhibitors, transporters, and importantly, virulence factors. The latest developments in understanding lipoproteins and their synthesis are described. A discussion is included about how this new information is being used for the development of vaccines and antimicrobial therapeutics. Finally, the collection concludes with an original research article, describing a structural analysis of acetylation sites among orthologs across multiple bacterial species and organisms (Jew et al.). The structures of five substrates of the lysine acetyltransferase YiaC were examined in-depth. Comparison of hundreds of structures in the Protein Data Bank (PDB) ultimately revealed inconsistencies in conclusions about lysine residue conservation in homologs based on primary linear sequence alone compared to the 3D structure. This highlights the need to consider where acetylation sites fall in three-dimensional space, when considering evolutionary conservation.

This special issue thus highlights original research and review articles on the topic of post-translational modifications in bacteria. They clearly illustrate the importance (and the challenge) of characterizing both the modified proteins and the enzymes involved in the addition/removal of PTMs, and of determining the impact of the modified residues on bacterial physiology (resistance, virulence, biofilm formation, etc.). All of these actors may be ultimately represent targets of interest to develop novel therapeutics to fight bacteria.

## Author Contributions

All authors listed have made a substantial, direct, and intellectual contribution to the work and approved it for publication.

## Conflict of Interest

The authors declare that the research was conducted in the absence of any commercial or financial relationships that could be construed as a potential conflict of interest.

## Publisher's Note

All claims expressed in this article are solely those of the authors and do not necessarily represent those of their affiliated organizations, or those of the publisher, the editors and the reviewers. Any product that may be evaluated in this article, or claim that may be made by its manufacturer, is not guaranteed or endorsed by the publisher.

